# Experimental detection of proteolytic activity in a signal peptide peptidase of *Arabidopsis thaliana*

**DOI:** 10.1186/1471-2091-14-16

**Published:** 2013-07-06

**Authors:** Masako Hoshi, Yu Ohki, Keisuke Ito, Taisuke Tomita, Takeshi Iwatsubo, Yoshiro Ishimaru, Keiko Abe, Tomiko Asakura

**Affiliations:** 1 Department of Applied Biological Chemistry, Graduate School of Agricultural and Life Sciences, The University of Tokyo, 1-1-1 Yayoi, Bunkyo-ku, Tokyo 113-8657, Japan; 2 Department of Neuropathology and Neuroscience, Graduate School of Pharmaceutical Sciences, The University of Tokyo, 7-3-1 Hongo, Bunkyo-ku, Tokyo 113-0033, Japan; 3 Department of Food and Nutritional Sciences, Graduate School of Nutritional and Environmental Sciences, University of Shizuoka, 52-1 Yada, Suruga-ku, Shizuoka 442-8802, Japan; 4 Core Research for Evolutional Science and Technology, Japan Science and Technology Agency, 7-3-1 Hongo, Bunkyo-ku, Tokyo 113-0033, Japan; 5 Department of Neuropathology, Graduate School of Medicine, The University of Tokyo, 7-3-1 Hongo, Bunkyo-ku, Tokyo 113-0033, Japan; 6 Food Safety and Reliability Project, Kanagawa Academy of Science and Technology, 3-2-1 Sakado, Takatsu-ku, Kawasaki 213-0012, Japan

**Keywords:** Signal peptide peptidase (SPP), Endoplasmic reticulum (ER), Aspartic protease, Regulated intramembrane proteolysis (RIP), *Arabidopsis thaliana*

## Abstract

**Background:**

Signal peptide peptidase (SPP) is a multi-transmembrane aspartic protease involved in intramembrane-regulated proteolysis (RIP). RIP proteases mediate various key life events by releasing bioactive peptides from the plane of the membrane region. We have previously isolated *Arabidopsis* SPP (AtSPP) and found that this protein is expressed in the ER. An AtSPP-knockout plant was found to be lethal because of abnormal pollen formation; however, there is negligible information describing the physiological function of AtSPP. In this study, we have investigated the proteolytic activity of AtSPP to define the function of SPPs in plants.

**Results:**

We found that an *n*-dodecyl-*ß*-maltoside (DDM)-solubilized membrane fraction from *Arabidopsis* cells digested the myc-Prolactin-PP-Flag peptide, a human SPP substrate, and this activity was inhibited by (Z-LL)_2_-ketone, an SPP-specific inhibitor. The proteolytic activities from the membrane fractions solubilized by other detergents were not inhibited by (Z-LL)_2_-ketone. To confirm the proteolytic activity of AtSPP, the protein was expressed as either a GFP fusion protein or solely AtSPP in yeast. SDS-PAGE analysis showed that migration of the fragments that were cleaved by AtSPP were identical in size to the fragments produced by human SPP using the same substrate. These membrane-expressed proteins digested the substrate in a manner similar to that in *Arabidopsis* cells.

**Conclusions:**

The data from the *in vitro* cell-free assay indicated that the membrane fraction of both *Arabidopsis* cells and AtSPP recombinantly expressed in yeast actually possessed proteolytic activity for a human SPP substrate. We concluded that plant SPP possesses proteolytic activity and may be involved in RIP.

## Background

Signal peptide peptidase (SPP) is a multi-transmembrane aspartic protease that contains two catalytic aspartates; the conserved YD and GXGD motifs in the sixth and seventh transmembrane domains, respectively [1]. SPP is located in the endoplasmic reticulum (ER) and the substrates of SPP are Type II membrane proteins, in which the locations of the N- and C-termini of these substrate proteins are in the cytosol and lumen, respectively. SPPs have been identified in human [1], mouse [2], zebrafish [3], fruit fly [4], *Caenorhabditis elegans*[5], *Arabidopsis thaliana*[6] and *Oryza sativa*[7]. Recessive lethal mutation studies of SPP in *Drosophila* indicated that SPP is necessary for development [4] and an SPP knockdown in zebrafish resulted in cell death in the central nervous system [3]. Moreover, a SPP knockdown in *C. elegans* led to embryonic death and an abnormal molting phenotype [5]. These data indicate that the SPP family is indispensable for survival. SPP appears to be involved in regulated intramembrane proteolysis through the cleavage of the substrate intramembrane region.

SPP promotes the intramembrane proteolysis of signal peptides following the cleavage of newly synthesized secretory or membrane proteins [8,9]. The resultant peptide fragments act as bioactive peptides that are liberated from the ER membrane. For example, the signal peptide fragments of human leukocyte antigens (HLA)-A liberated by SPP bind to HLA-E molecules and are subsequently presented to NK-cells for immune surveillance [10,11].

The secretory protein hormone preprolactin is also processed by SPP and the resulting N-terminal fragments are released into the cytosol [8]. Thereafter, the fragments bind to calmodulin and enter into the cellular signal transduction pathway [12]. In addition, SPP participates in the maturation of the core protein of the hepatitis C virus (HCV) [13]. SPP also possesses non-enzymatic functions, including molecular chaperone activity. SPP interacts with the human cytomegalovirus glycoprotein US2 and induces the dislocation of MHC class I heavy chains to the proteasome system [14]. Moreover, based on the observation that SPP interacts with newly synthesized membrane proteins *in vitro*, human SPP (HsSPP) interacts with signal peptides and misfolded membrane proteins that are removed during ER quality control. However, SPP does not interact with all types of membrane proteins [15]. Thus, the function of mammalian SPP has been examined, yet there are only a few studies that have examined plant SPPs. We have previously isolated AtSPP (At2g03120) in *Arabidopsis thaliana*, and have shown that AtSPP is strongly expressed in the shoot meristem of germination seeds and in the inflorescence meristem during the reproductive stage [6]. We have investigated a GFP-fused AtSPP protein in cultured “Deep” cells and found that this protein is localized in the ER. Moreover, subcellular localization studies of endogenous AtSPP in “Deep” cells by equilibrium sucrose density gradient centrifugation also indicated that AtSPP is localized in the ER [6]. The shoot meristem contains undifferentiated cells that have the potential to differentiate into all aerial parts of the plant [6]. Based on these results, it is conceivable that AtSPP is involved in regulating growth and differentiation in *Arabidopsis*. This notion is supported by a knockout of the AtSPP gene giving rise to a lethal phenotype [16]. However, the target molecule of SPP in plants remains unresolved. Recently, it was reported that nodule-specific cysteine rich polypeptides (NCR) mediate consecutive differentiation events with symbiosomes in *Medicago truncatula*[17]. NCR propeptides are likely to be processed by the signal peptidase complex (SPC) and converted to the active form. The highly correlated expression of SPC with SPP suggests that NCR signal peptides can be processed by SPP [18,19]. To reveal the function of plant SPPs, it is important to examine the proteolytic activity of SPPs.

Herein, we present evidence that the SPP of *Arabidopsis* actually possesses proteolytic activity, suggesting that the plant SPP cleaves the target proteins in the membrane and releases bioactive peptides that function in signal transduction pathways, similar to the phenomena observed for other species.

## Results

### Preparation of the membrane fraction of “deep” cell extracts and the proteolytic activity of this fraction

**Figure 1:**
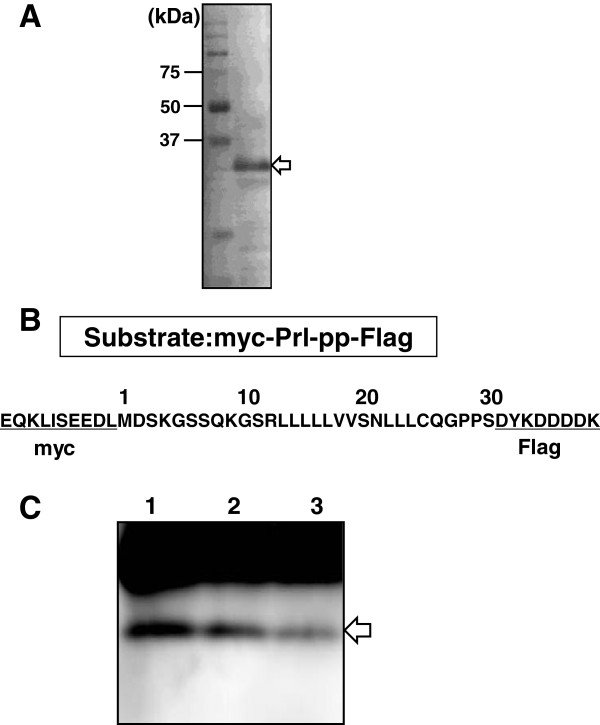
**Proteolytic activity of the membrane fraction of “Deep” cell extracts. (A)** Immunoblot analysis of the DDM solubilized membrane fraction of “Deep” cells. The location of the AtSPP protein is indicated by the white arrow. **(B)** Structure of the substrate: the signal sequence of the bovine prolactin mutant (Prl-pp) fused with a myc epitope tag at the N-terminus and a Flag tag epitope at the C-terminus. **(C)** “Deep” cell membrane fractions solubilized with 0.25% DDM were incubated for 3 h in the presence of the inhibitor. Lane 1: control (without inhibitor); lane 2: 10 μM (Z-LL)_2_-ketone; and lane 3: 20 μM L-685,458. The arrow indicates the proteolytic products from the myc-Prl-PP-FLAG peptide detected with an anti-myc antibody.

For the purposes of studying the proteolytic activity of AtSPP, we have isolated the membrane fraction of *Arabidopsis* root derived cultured “Deep” cells. The AtSPP protein was detected by SDS-PAGE as a single band in the membrane fraction of “Deep” cells (Figure 1A). The deduced size of the protein estimated from the primary sequence was 38 kDa. The band representing AtSPP migrated further on the SDS-PAGE than the estimated molecular weight. Nonetheless, such anomalous electrophoretic migration has been shown previously for ER fractions isolated from “Deep” cells [6].

Several detergents were tested for their suitability to solubilize active AtSPP from the membranes. Digitonin, CHAPS-, CHAPSO- and NP-40-solubilized membrane fractions were examined for their activity to digest the synthetic peptide myc-Prl-PP-Flag [20] (Figure 1B). Although all of these membrane fractions showed proteolytic activities, none were inhibited by the SPP-specific inhibitor (Z-LL)_2_-ketone (data not shown). This indicates that the proteolytic activity of the membrane fraction was induced by proteases other than SPP. We then tested whether an *n*-dodecyl-*ß*-maltoside (DDM)-solubilized membrane fraction showed proteolytic activity, because human SPP has been shown to exhibit proteolytic activity using this preparation [20]. As shown in Figure 1C, this fraction (0.079 μg of protein) actively cleaved the myc-Prl-PP-Flag peptide and was inhibited by (Z-LL)_2_-ketone, as well as L-685,458, an aspartic protease inhibitor that targets SPP or presenilin [21]. Based on these results, we concluded that the DDM-solubilized membrane fraction possesses SPP-like proteolytic activity, and likely has activity from other proteases.

### AtSPP-GFP fusion protein expression in *Saccharomyces cerevisiae*

**Figure 2:**
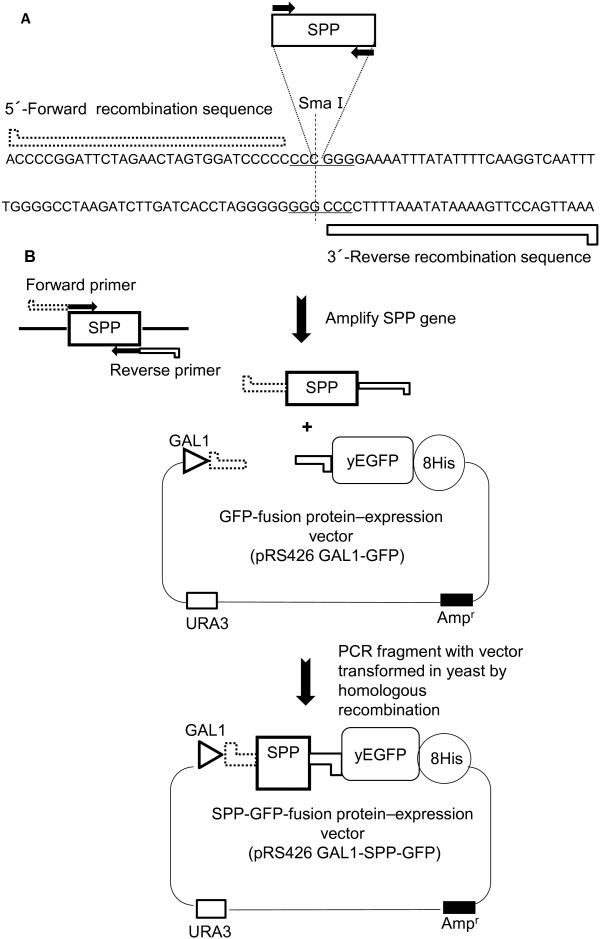
**Construction of GFP-fusion protein expression plasmid. (A)** Cloning site of pRS426 GAL1-GFP and overlapping recombination sequence of the primer. **(B)** SPP genes were amplified with primers described in the Materials and Methods. The *Sma*I digested vector and PCR products were transformed into *Saccharomyces cerevisiae* by homologous recombination. yEGFP stands for enhanced green fluorescent protein.

**Figure 3:**
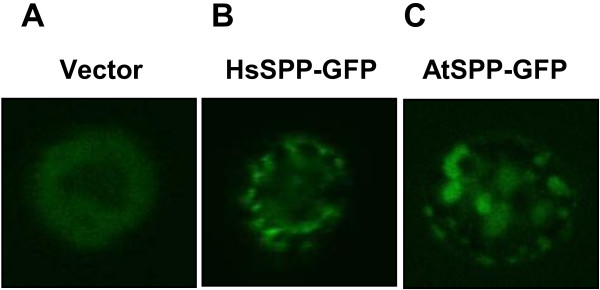
**SPP-GFP fusion protein expression in yeast as visualized by confocal microscopy.** An aliquot of the yeast culture cell suspension overexpressing the fusion protein was placed onto a microscope slide to observe GFP fluorescence by microscopy. **(A)** Fluorescence from the control cells harboring only the vector. GFP fluorescence was not detected. **(B)** Transformation with HsSPP-GFP. **(C)** Transformation with AtSPP-GFP.

**Figure 4:**
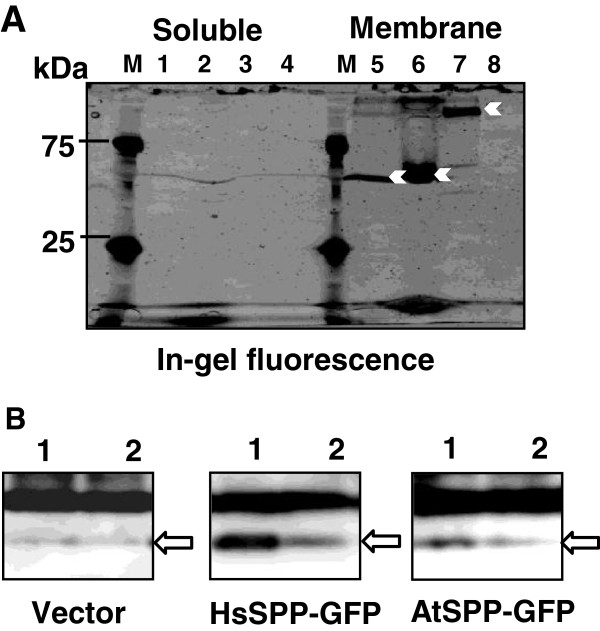
**Expression of the SPP-GFP fusion protein in yeast. (A)** Soluble and membrane fractions were analyzed by 12% SDS-PAGE using in-gel fluorescence. The protein concentrations loaded were as follows: Soluble fractions in the left panel; lane 1, AtSPP-GFP, 117 μg; lane 2, AtSPP-GFP, 340 μg; lane 3, HsSPP-GFP, 56 μg; and lane 4, vector, 56 μg. Membrane fractions in the right panel: lane 5, AtSPP-GFP, 306 μg; lane 6, AtSPP-GFP, 1120 μg; lane 7, HsSPP-GFP, 158 μg; and lane 8, vector, 161 μg. Detection of GFP fluorescence in the gel was carried out by exposure to blue light (EPI) at 460 nm with a cut-off filter. The arrows indicate the location of the GFP fusion proteins. **(B)** Proteolytic activity of the membrane fraction of GFP-fusion protein. Solubilized membrane fractions of yeast cells overexpressing the vector control (183.1 μg) HsSPP-GFP (197.5 μg) and AtSPP-GFP (55 μg) proteins were incubated for 15 h in the presence or absence of the inhibitor. Lane 1: control (without inhibitor); and lane 2: 50 μM (Z-LL)_2_-ketone. Arrows indicate the proteolytic products from myc-Prl-PP-FLAG detected with the anti-myc antibody.

To determine whether the proteolytic activity of the DDM-solubilized “Deep” cell membrane fraction was indeed caused by AtSPP, we expressed an AtSPP GFP-fusion protein (AtSPP-GFP) in yeast cells, as described previously [22]. As shown in Figure 2, the linearized vector (pRS426- GAL1-GFP) and amplified PCR products were transformed into *S. cerevisiae* BY2777. Figure 3 shows the confocal microscopy image of HsSPP-GFP and AtSPP-GFP localization. Yeast cells transformed with the vector alone did not exhibit any GFP fluorescence; however, fluorescence was detected in the HsSPP-GFP- and AtSPP-GFP-transformed cells. These results indicate that the AtSPP-GFP fusion protein was successfully expressed. We next confirmed the expression of the fusion proteins by in-gel fluorescence. Yeast cells were separated into soluble and membrane-bound fractions after mechanical disruption. Fluorescent bands of GFP-fusion proteins are shown in Figure 4A. No band was detected in the soluble fraction. The 2% DDM-solubilized membrane fraction band is indicated by the white arrowheads. Moreover, the intensity of the AtSPP-GFP band increased in a dose-dependent manner (Figure 4A). These data suggest that AtSPP-GFP and HsSPP-GFP are localized in the yeast membrane fraction. Based on their primary sequences, the sizes of AtSPP-GFP and HsSPP-GFP are estimated to be 65 and 72 kDa, respectively. AtSPP-GFP migrated at a size smaller than 65 kDa, whereas HsSPP-GFP migrated at a size more than 72 kDa. While the reasons underlying this anomalous migration remain to be elucidated, previous Blue Native Polyacrylamide Gel Electrophoresis (BN-PAGE) studies have shown that HsSPP forms a high molecular mass complex under DDM-solubilized conditions [23]. HsSPP and AtSPP may assemble into complexes of different molecular masses.

### *In vitro* cell-free assay using AtSPP overexpressed in yeast

Next, the membrane fractions containing GFP-fusion proteins were examined for proteolytic activity using an *in vitro* assay system. Both HsSPP-GFP and AtSPP-GFP exhibited proteolytic activity in the *in vitro* assay. Faint bands were detected in the lanes of the vector control. However, this band was not inhibited by the (Z-LL)_2_-ketone. This cleavage was due to an intrinsic proteinase present in yeast. Though the rate of proteolytic activity of AtSPP-GFP was weak, the activity was inhibited by the (Z-LL)_2_-ketone, an SPP-specific inhibitor (Figure 4B). Our results suggest that the AtSPP-GFP fusion protein possesses proteolytic activity.

**Figure 5:**
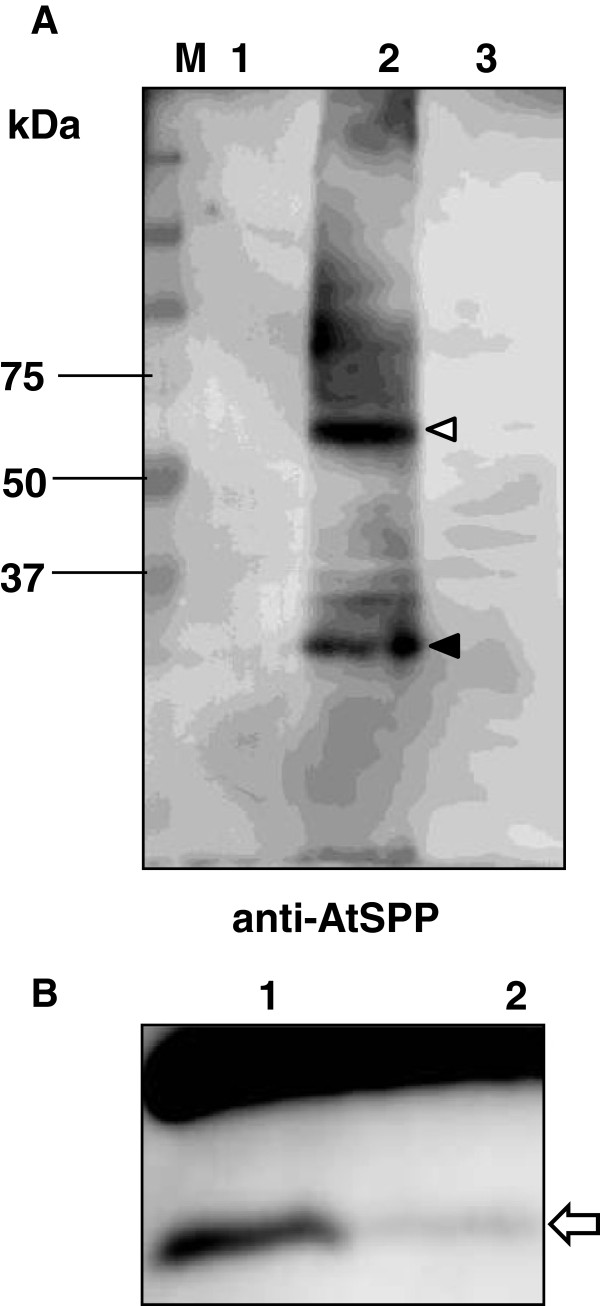
**Identification of the proteolytic activity by overexpressing AtSPP in yeast. (A)** Immunoblot analysis of DDM-solubilized membrane fraction overexpressing AtSPP in yeast by using anti-AtSPP. Lane 1, vector control; lane 2, overexpression of AtSPP in yeast; and lane 3, overexpression of HsSPP-GFP in yeast. The filled-arrowhead indicates the monomer and the open-arrowhead indicates the dimer. **(B)** Evaluation of the proteolytic activity of AtSPP overexpressed in yeast. Proteolytic activity of the membrane fraction of yeast cells overexpressing AtSPP with inhibitor. Lane 1: control (without inhibitor); lane 2: 1 μM(Z-LL)_2_ small capitor-ketone. The arrow indicates the proteolytic products from myc small capitor-Prl-PP-FLAG peptide detected with the anti-myc antibody.

To confirm the proteolytic activity of AtSPP, we expressed AtSPP without the GFP protein, thereby creating a protein construct that is closer to the native form. DDM-solubilized yeast membrane fractions were extracted and AtSPP was detected by immunoblotting using an anti-AtSPP antibody (Figure 5A). The arrowheads indicate the bands which specifically cross-reacted with the anti-AtSPP antibody. The overexpressed AtSPP detected in yeast was identified in the gel analysis by two bands. Electrophoretic migration of AtSPP in “Deep” cells (Figure 1A) matched the lower band detected in the yeast sample. Therefore, the lower molecular weight band may be a monomeric species and the higher molecular weight band may represent a dimer that is not affected by SDS treatment.

No bands were detected in the control vector (Figure 5A, lane 1) and the HsSPP-GFP-transformed cell samples (Figure 5A, lane 3). The proteolytic activity of AtSPP in the presence of the (Z-LL)_2_-ketone is shown in Figure 5B. The proteolytic activity toward myc-Prl-PP-FLAG is almost completely inhibited by 1 μM (Z-LL)_2_-ketone. In summary, our results confirm that AtSPP proteolytically cleaves the myc-Prl-PP-FLAG substrate *in vitro*.

### Comparison of the cleavage site of myc-Prl-PP-FLAG using electrophoresis

**Figure 6:**
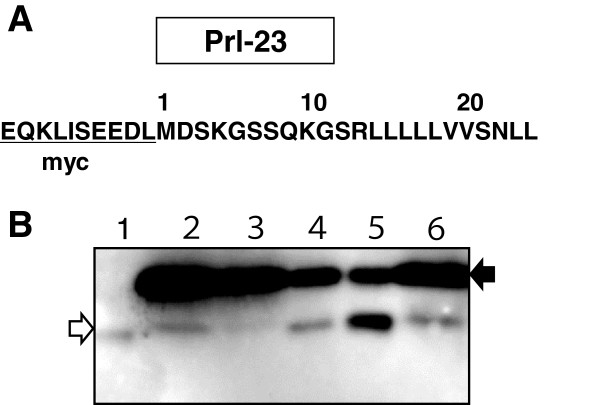
**Characterization of the cleavage site in the myc-Prl-PP-FLAG peptide.** Products of the myc-Prl-PP-FLAG peptide proteolysis by various SPP proteins and standard peptides Prl-23 were separated by a Tris/Tricine urea gel and detected with an anti-myc antibody. **(A)** Structure of the substrate Prl-23: Prl-23 is a N-terminal fragment of myc-Prl-PP-FLAG peptide composed of 23 amino acid residues. **(B)** Lane 1, synthesized marker fragment of Prl-23; lane 2, the membrane fraction of HEK293T cells; lane 3, HEK293T cells with 10 μM (Z-LL)_2_-ketone inhibitor; lane 4, membrane fraction of yeast cells overexpressing HsSPP-GFP; lane 5, membrane fraction of “Deep” cell extracts; lane 6, the membrane fraction of yeast cells overexpressing AtSPP. The black arrow indicates myc-Prl-PP-FLAG as the original peptide and the white arrow indicates the position of the proteolytic fragment.

Electrophoresis of the fragments from myc-Prl-PP-FLAG was carried out and detected by an anti-myc antibody. A MALDI-TOF mass spectrometry study showed that human SPP cleaved primarily at a single site between Leu-23 and Leu-24 [17]. To compare the main cleavage sites, fragments from myc-Prl-PP-FLAG cleaved by various SPPs were co-electrophoresed with a synthesized marker fragment of Prl-23 (Figure 6A) on a Tris/Tricine urea gel. Figure 6 shows that HEK293T cells expressing native human SPP cleaved myc-Prl-PP-FLAG at the C-terminal of Leu-23 and the SPP was inhibited by the (Z-LL)_2_-ketone. The fragment arising from the digestion of the synthetic Prl-23 by the overexpressed HsSPP-GFP fusion protein, membrane fraction of “Deep” cells and recombinant AtSPP migrated to the same location (Figure 6B). This result indicates that AtSPP cleaved mainly myc-Prl-PP-FLAG at the same sequence as the human SPP did.

## Discussion

We have detected the AtSPP protein in a membrane fraction of “Deep” cells (Figure 1A). The proteolytic activity of the DDM-solubilized “Deep” cell membrane fraction was blocked by the (Z-LL)_2_-ketone and L-685,458 inhibitors (Figure 1C). These results confirm that SPP exists in the membrane fraction of “Deep” cells; however, the result also indicates that an aspartic protease other than SPP exists in this fraction. Proteolytic activity of SPP was detected in the DDM-solubilized membrane fraction, but not in the other detergent-solubilized membrane fractions prepared. Previous studies have shown that the γ-secretase complex dissociates in DDM [24] and thus, γ-secretase should not be active in the DDM solubilized membrane fraction. Therefore, the difference in the extent of inhibition brought about by (Z-LL)_2_-ketone and L-685,458 may be due to the presence of an aspartic protease other than γ-secretase. AtSPP and AtSPP-GFP fusion proteins were successfully sorted toward the membrane fraction and were expressed in *S. cerevisiae*. Moreover, expressed AtSPP was observed to cleave the substrate myc-Prl-PP-Flag.

We have observed proteolytic activity of endogenous SPP in human HEK293T cells (Figure 6B). Similarly, the proteolytic activity of the HsSPP-GFP fusion protein overexpressed in yeast was found to efficiently process preprolactin, because the preprolactin sequence is derived from mammals. The signal sequences have some common features, although there are no apparent common sequences [25]. The signal sequence consists of a tripartite structure: a central hydrophobic h-region, a positively charged hydrophilic n-region and a C-terminal flanking polar region. The h-region often contains helix-breaking amino acids, such as glycine, proline, tyrosine and serine. The central hydrophobic h-region of the preprolactin signal sequence also has helix-destabilizing serine and asparagine residues. Although the preprolactin sequence is not a native substrate of the plant SPP, we have identified that AtSPP can cleave at the typical position after the helix-breaking amino acids in the central hydrophobic h-region, in a similar manner to that in which human SPP cleaves preprolactin.

Immunoblot analysis of the DDM-solubilized membrane fraction from the AtSPP-overexpressing yeast cells using an anti-AtSPP antibody detected two bands (Figure 5A). In contrast, the DDM-solubilized “Deep” cell membrane fraction extracted by the same method detected only one band (Figure 1A). Recently, Miyashita et al. [23] suggested that the proteolytic activity of *Drosophila* SPP requires its assembly into high molecular weight complexes. HsSPP was also reported to assemble into oligomeric complexes [26]. HsSPP can be isolated both as a monomer and as an SDS-stable dimer. Under denaturing conditions, AtSPP was isolated as a monomer in the “Deep” cell sample and as a SDS-resistant dimer when overexpressed in yeast. Thus, it is possible that AtSPP may assemble into both oligomeric complexes and monomer under native conditions.

A previous paper showed that the sequence homology around the active site motifs YD, GLGD and PAL is highly conserved [1]. In contrast, the sequence of the N-terminus of AtSPP and the transmembrane region 1 (TM1) differs noticeably when compared with the sequences from other species. Although the topological conformation to face the membrane is reversed, the active site and PAL motif of human SPP and presenilin are identical, suggesting a common catalytic mechanism. The structural analysis of the initial substrate binding site by γ-secretase modulators (GSMs) showed GSMs bind directly to the TM1 of PS and affect the structure of the catalytic site [27]. Human SPP was also affected by non-steroidal anti-inflammatory drugs (NSAIDs ) that function as GSMs [20]. These data suggest a crucial role of the TM1 in the intramembrane cleaving mechanism. It is very interesting that AtSPP possessing a different TM1 cleaved the same substrates as human SPP. Hereafter, unraveling how the digestion occurs by modification experiments in the TM1 region of AtSPP is required.

Currently, the native substrates targeted by SPP in plants have not been identified. Further investigation of these substrates is needed for understanding the function of SPPs in plants. Research detailing the native substrates and the role of SPPs in the plant life cycle is in progress.

## Conclusion

“Deep” cells possess a signal peptide peptidase with proteolytic activity.

AtSPP and AtSPP-GFP were expressed in the membrane fraction of *S. cerevisiae* and shown to digest the HsSPP substrate. The activities of AtSPP and AtSPP-GFP were inhibited in the presence of an SPP-specific inhibitor. The main cleavage site of AtSPP was identified as identical to the site in human SPP. We conclude that plant SPPs possess proteolytic activity, and that this activity is likely to be involved in RIP.

## Methods

### Materials and cell culture

Myc-Prl-PP-Flag [28] was synthesized by BEX Co., LTD. (Tokyo, Japan) with the sequence illustrated in Figure 1B. Prl-23 was also synthesized by BEX Co., LTD. (Tokyo, Japan) with the sequence illustrated in Figure 6A. [(2*R*,4*R*,5*S*)-2-Benzyl-5-(*t*-butyloxycarbonylamino)-4-hydroxy-6-phenylhexanoyl]-L-leucyl-L-phenylalanine amide (L-685,458) [29] and 1,3-di-(*N*-carboxybenzoyl-l-leucyl-l-leucyl) amino acetone ((Z-LL)_2_-ketone) [30] were purchased from Calbiochem (San Diego, CA. USA) and PEPTIDE INSTITUTE INC. (Osaka, Japan), respectively.

*Arabidopsis* root cells (“Deep” cells) were cultured at 22°C in Murashige and Skoog medium under dark conditions. The rabbit polyclonal anti-AtSPP C terminus antibody was obtained as described previously [6]. The rabbit anti-c-myc polyclonal antibody was purchased from Sigma-Aldrich (St. Louis, MO, USA).

### Extraction of membrane fractions and immunoblotting

The extraction of membrane fractions was carried out as described previously [20]. Briefly, a “Deep” cell suspension culture (100 ml) was centrifuged and the pellet was collected. The cells were homogenized in (H) buffer (50 mM HEPES, pH 7.0, 250 mM sucrose, 5 mM EDTA) containing a complete protease inhibitor cocktail (Roche, Basel, Switzerland). The cells were disrupted using a French press at 1,000 psi and centrifuged at 3,000 × *g* for 10 min to remove cell debris and nuclei. The supernatant was centrifuged again at 100,000 × *g* for 60 min to isolate the microsomal fraction. Microsome pellets were resuspended in 2% DDM-containing (H) buffer for 90 min on ice, and then centrifuged at 25,000 × *g* for 15 min. The solubilized membrane fraction was passed through an Amicon Ultra-0.5 centrifugal filter device-10 K (Millipore, Tokyo, Japan) and then diluted for the assay. Yeasts were cultured at 30°C for 22 h after induction by galactose. The cells were collected and harvested using a Multi-beads shocker (Yasui Kikai, Osaka, Japan). Human embryonic kidney (HEK) 293 T cells were incubated in Dulbecco’s modified Eagle’s medium (DMEM, Sigma Aldrich, St. Louis, MO, USA) supplemented with 10% fetal bovine serum (Invitrogen, Carlsbad, CA, USA) at 37°C under 5% CO_2_ and were collected and harvested using a French press at 1,000 psi. The membrane fractions of yeasts and HEK 293 T cells were obtained in a similar manner to the process used for *Arabidopsis* cells after harvesting. SDS-PAGE and immunoblotting were performed as described previously [6].

### Expression of GFP-fusion SPP in *Saccharomyces cerevisiae*

The GFP-fused protein-expression vector (pRS426 GAL1-GFP) [22] was kindly provided by Dr. Iwata (Imperial College, London). The *S. cerevisiae* BY2777 (MATa prb1-1122 prc1-407 pep4-3 ura3-52 leu2 trp1) strain was provided by the National Bio-Resource Project (NBRP), MEXT, Japan. This plasmid was composed of a C-terminal yeast-enhanced green fluorescent protein (yEGFP), which is lacking the N-terminal methionine, fused with an octa-His tag, and harbors a GAL1 promoter and URA selection marker (Figure 2). The SPP protein sequence, lacking a stop codon, was inserted into the reverse primer for GFP expression. DNA was amplified with the following primer pair: HsSPP forward primer (5′-accccggattctagaactagtggatcccccatggactcggccctcagcgatc-3′) and reverse primer (5′-aaattgaccttgaaaatataaattttcccctttctctttcttctccagccccttc-3′). AtSPP-GFP forward primer (5′-accccggattctagaactagtggatcccccatgaagaattgtgagagatttgc-3′) and reverse primer (5′-aaattgaccttgaaaatataaattttccccttcatcatgagctttattaacctc-3′). For studying the expression of the protein without GFP, another reverse primer was prepared, the AtSPP reverse primer, (5′-aaattgaccttgaaaatataaattttcccctcattcatcatgagctttattaacc-3′) (Figure 2).

The expression plasmids were constructed as follows: the expression vector (pRS426 GAL1-GFP) was linearized with *Sma*I and the amplified fragment was inserted by homologous recombination using Frozen-EZ Yeast Transformation II (Zymo Research, Irvine, CA, USA). The transformants were selected by growing in the absence of the uracil medium, as described previously [22]. The expression of SPP-GFP was confirmed by confocal microscopy (FV10i-LIV, Olympus Tokyo Japan). In-gel fluorescence was performed using an Image Quant LAS-4000mini (GE Healthcare, Amersham, Uppsala, Sweden).

### *In vitro* cell-free assay

Membrane fractions solubilized by 0.25% DDM were incubated at 37°C with 1 μM of the myc-Prl-PP-FLAG peptide (BEX Co., Ltd., Tokyo, Japan) containing a protease inhibitor cocktail (Roche, Basel, Switzerland) for the appropriate times. For the inhibitor assay, the reaction mixtures were incubated in the presence or absence of the (Z-LL)_2_-ketone), a SPP inhibitor, and L-685,458, an aspartic protease inhibitor. Dimethyl sulfoxide was used as a vehicle control. Products were separated on a 15% Tris/Tricine SDS gel containing 8 M urea, then transferred to a 0.2-μm polyvinylidene difluoride membrane (Whatman, Maidstone, UK), and detected with an anti-c-myc antibody. Signal detection was performed with the Image Quant LAS-4000mini (GE Healthcare) using the ECL system (GE Healthcare).

## Abbreviations

SPP: Signal peptide peptidase; ER: Endoplasmic reticulum; RIP: Regulated intramembrane proteolysis; HEK: Human embryonic kidney; CBB: Coomassie brilliant blue; DDM: *n*-dodecyl-ß-maltoside; NK-cell: Natural killer cells; MHC: Major histocompatibility complex; HLA: Human leukocyte antigens; NCR: Nodule-specific cysteine rich; SPC: Signal peptidase complex; GFP: Green fluorescent protein; GSMs: γ-secretase modulators; NSAIDs: Non-steroidal anti-inflammatory drugs; PS: Presenilin.

## Competing interests

The authors declare that they have no competing interests.

## Authors’ contributions

MH, KA and TA designed the research. MH performed all the research. YO and KI constructed the enzyme assay and yeast expression system, respectively. TT analyzed the data. MH, YO, KI, TT, TI, YI, KA and TA wrote the paper. All authors read and approved the final manuscript.
